# Electrophysiological measurements with electrode types of different perimodiolar properties and the same cochlear implant electronics – a retrospective comparison study

**DOI:** 10.1186/s40463-019-0361-8

**Published:** 2019-09-06

**Authors:** A. Perenyi, F. Toth, B. Dimak, R. Nagy, P. Schoerg, J. Jori, J. G. Kiss, G. Sprinzl, M. Csanady, L. Rovo

**Affiliations:** 1 Department of Otorhinolaryngology, Head and Neck Surgery, University of Szeged, Tisza Lajos krt. 111, Szeged, H-6725 Hungary; 2 Karl Landsteiner University Hospital of StPölten, Propst-Führer-Straße 4, 3100 St. Pölten, Austria

**Keywords:** Cochlear implant, CI532, CI512, CI522, Electrode array, Perimodiolar, Electrophysiology

## Introduction

There are currently different trends in cochlear implant electrode design [1]. The manufacturers provide a variety of implant configurations including different receiver-stimulators, electrode arrays (e.g. straight or pre-curved, full-length or short) and sound processors to choose from, which can facilitate decision making on an individual basis. Proximity to the modiolus [2, 3], electrical current requirements [4], energy consumption, trauma to the cochlea [5], combined electro-acoustic stimulation [6, 7]), preservation of cochlear structures with low-trauma surgical technique [3, 8–10] and hearing preservation [11–14] are important aspects of implant design which have become the focus of many discussions and studies.

For example, recent evidence suggests that speech discrimination is not improved by deep insertion, but it is significantly improved by perimodiolar position of the electrode [15].

Studies in implanted recipient groups using multiple implant types make it difficult to compare the influence of the implant electrode characteristics on outcomes in the presence of additional variables such as implant electronics, sound processors and speech coding paradigms. Hence, to reduce the number of variables, comparison of the influence of electrode designs on outcomes could be interpreted more effectively if a consistent receiver-stimulator design and a common sound processor are used. Recent publications [16–20] represent imaging and electrophysiological results with CI532, but no comparative studies have yet been published.

Our center’s postoperative radiological comparative study demonstrated that the Slim Modilar electrode array took a closer position to the modiolus than the Contour Advance electrode array [21].

As a consequence, the authors’ aim in this multicenter study that is to their knowledge the first with this focus was to compare the influence of various electrode designs upon selected electrophysiological outcomes for cochlear implant recipients using the same model of receiver-stimulator, Cochlear™ Nucleus® Profile Series and sound processor in a retrospective study.

## Materials and methods

### Inclusion and allocation of subjects

A total of 139 consecutive subjects who were implanted between 13 June 2014 and 4 May 2017 with a Profile CI532 (CI532), a Profile CI512 (CI512), and a Profile CI522 (CI522) device manufactured by Cochlear Ltd., Australia and gave their informed consent were recruited to this retrospective study from two tertiary referral implant centers. Time periods of the study recruitment were from 13 June 2014 to 14 December 2015 for CI512, from 13 November 2015 to 4 May 2017 for CI532and 11 March 2015 to 29 November 2016 for CI522. All subjects were examined with high resolution computed tomography and/or magnetic resonance imaging before surgery. Exclusion criteria were cochlear malformations, cochlear otosclerosis, obliterative postmeningitis changes and electrode tip foldover. To the authors knowledge there were no neural disorders in either group. Postoperative radiography was performed in each subject to confirm that the active electrode occupied an intracochlear position with no complications or abnormal electrode position.

The subjects were allocated into groups based on the electrode type implanted as shown in Table 1. Those who received a CI532 formed group 532, those who received a CI512 formed group 512, and those who received a CI522 formed group 522. Subjects were consecutively treated as part of routine clinical practice that was comparable at each respective implant site.

- A total of 159 ears in 139 subjects were implanted with devices, including the same implant receiver-stimulator electronics. CI532 had a 22 electrode array which was perimodiolar and with a relatively smaller diameter (named Slim Modiolar), CI512 had a 22 electrode array which was perimodiolar with a relatively larger diameter (named Contour Advance), and CI522 had a 22 electrode array which was straight, also with a relatively small diameter (named Slim Straight). A total of 54 ears were implanted with CI532 (all in Clinic 1), 54 ears with CI512 (51 in Clinic 1 and 3 in Clinic 2), and 51 ears with CI522 (47 in Clinic 2 and 4 in Clinic 1). Patients who were implanted with CI532 formed the test group. Two control groups were formed from patients who were implanted with Implants 512 and 522. The underlying causes of hearing loss were congenital, progressive, unknown and others (e.g. choesteatoma, infection, Meniere’s disease, meningitis, ototoxic drugs, sudden hearing loss, trauma) in 29, 22, 16, and 33% for group 532, 28, 26, 28, and 17% for group 512, and 17, 23, 35, and 25% for group 522, respectively.

**Table 1:** Subject demographics for each subject group. Note: For continuous variables, the mean and + 1 standard deviation are shown in brackets

Subject group	532	512	522
Device	CI532	CI512	CI522
Electrode type	Slim modiolar	Contour advance	Slim straight
Number of patients	46	45	48
Number of ears	54	54	51
Age (year)	25.17±26.29	20.80±25.87	55.36±28.59
Sex (male/female)	25/29	23/31	33/18
Duration of deafness (year)	2.94±7,46	3.06±9.34	3.13±12.99
Cause of deafness			
Congenital	29%	28%	17%
Progressive	22%,	26%	23%
Unknown	16%	28%	35%
Others	33%	17%	25%

### Implantation technique

The electrode arrays were inserted into the cochlea according to the manufacturer’s instructions provided in the physician’s surgical guide. The method of electrode insertion was identical in both implant clinics [22]. Full insertion was achieved via the extended round window approach with CI532 and CI512 and via the round window approach with CI522 in all ears. The AOS (advance off-stylet) technique was used for CI512 and the freehand technique was used for CI522. Electrode choice was dependent on the actual implant pool of each center (regulated by the health authorities). The age of the patients did not influence implant choice. Discussion of hearing preservation was not an objective of this study.

### Electrophysiological testing

The three different types of electrode arrays were compared with regards to outcomes from intraoperative and 3-months postoperative electrophysiological testing performed as per routine clinical protocol (Table 2).

**Table 2:** Summary of the intraoperative and postoperative evaluation protocols and available data sets for each type of electrode. The routine protocol in Clinic 2 did not include measurement of intraoperative ESRT, and postoperative T-NRT

	Group 532 Nucleus CI532 (n/54 implants)	Group 512 Nucleus CI512 (n/54 implants)	Group 522 Nucleus CI522 (n/51 implants)
intraoperative ESRT	44	47	0
intraoperative T-NRT	50	47	43
postoperative C-level (1 month)	54	54	51
postoperative C-level (3 month)	54	54	51
postoperative T-NRT (3 month)	32	36	0

Intraoperative electrophysiological tests were carried out as part of the regular fittings with Nucleus Custom Sound 4.4 software: Impedance was measured for each electrode, the electrical stapedial reflex threshold (ESRT) with 25 μs pulse width for every second electrode contact (No. 2, 4, 6 etc.) and neural response telemetry threshold (T-NRT) for 6 (No. 2, 6, 10, 14, 18 and 22) electrode contacts. ESRT values were compared in groups 532 and 512. T-NRT values in group 532 were compared with those in both control groups. A common sound processor (Nucleus CP910) was used.

The centers followed their normal routine protocol, thus the electrophysiological measurement protocol of the two centers was not identical, i.e. intraoperative ESRT testing, postoperative T-NRT measurements were not included in the routine protocol by Clinic 2, and thus CI522 was not analyzed with regards to these parameters. Furthermore, postoperative NRT was not measured for subjects in each group, where the current required to elicit a threshold response exceeded their discomfort or pain level.

The first fitting was performed 4 weeks after surgery in each case. In order to determine the electric threshold (T-levels), and comfort threshold (C-levels), the subjective fitting method was used in adults and the semi-objective NRT based fitting (based on the intraoperative T-NRT results) was applied in children [23, 24]. Default MAP parameters (25 μs pulse width, 900 Hz stimulation rate and 8 maxima) were used. Postoperative NRT was measured 2 months after the first fitting, i.e. 3-months follow-up. C-levels at first fitting and 3-months follow-up fitting and T-NRT at 3-months follow-up were compared.

Outcomes for precurved slim perimodiolar electrode design, used at one implant clinic were compared to outcomes for two control groups of recipients implanted with precurved perimodiolar and straight electrodes in both implant clinics. Electrode designs were compared on the basis of outcomes for intraoperative objective electrophysiological measures and postoperative threshold levels and comfort levels to characterize electrode position within the cochlea.

### Statistical analysis

Statistical analysis with the Student’s t-test (*P* < 0.05) and one-way repeated measures ANOVA test were performed with 95% confidence interval (*p* < 0.05). Before the calculation, tests for normality of data distribution were performed. Bonferroni correction was used as needed to consider multiple variables (e.g. comparison of all three implant groups). The comparison was made on each electrode and all of the electrodes (Grand average). The tests were performed with Microsoft Excel 2016 and SPSS for Windows.

## Results

All subjects received Nucleus Profile implants. The only difference was the type of electrode. The patient groups were similar in subject numbers, etiology and duration of deafness, and indications.

### Electrophysiology testing

#### Intraoperative measurements

**Fig. 1:**
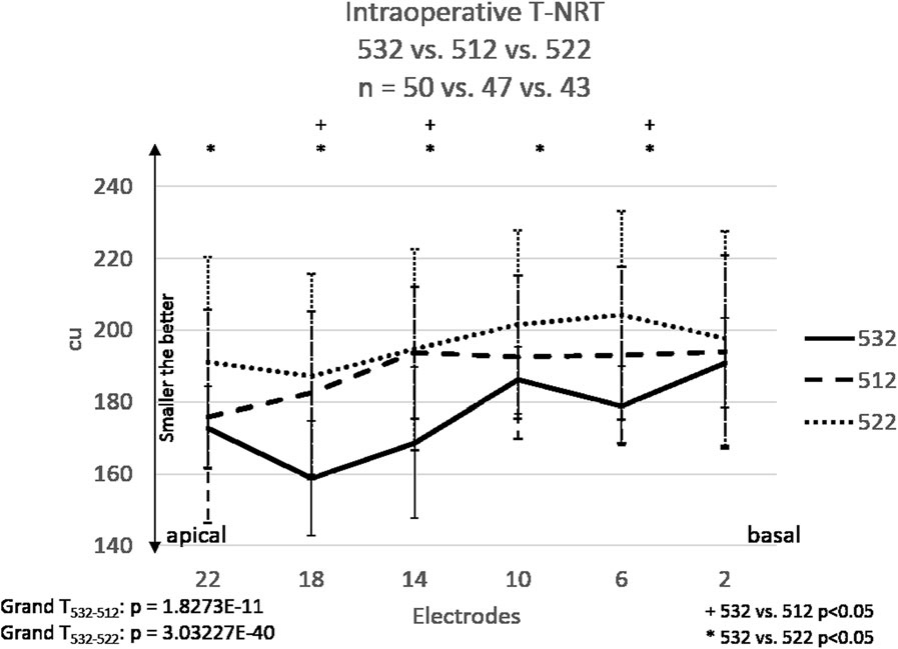
Intraoperative T-NRT values in all groups: Groups 532 (*n* = 50), 512 (*n* = 47) and 522 (*n* = 43). The “+” stands for significant difference between groups 532 and 512. The “*” represents a significant difference between groups 532 and 522. Error bars represent the standard deviation (SD). The mean NRTs proved to be lower in each electrode in group 532 when compared with both control groups. The difference was significant in 5 measured electrodes when compared with 522 and 3 measured electrodes when compared with 532 (t-probe: *p* < 0.05). Grand T_532–512_ means statistical comparison between groups 532 and 512. Grand T_532–522_ means statistical comparison between groups 532 and 522

**Fig. 2:**
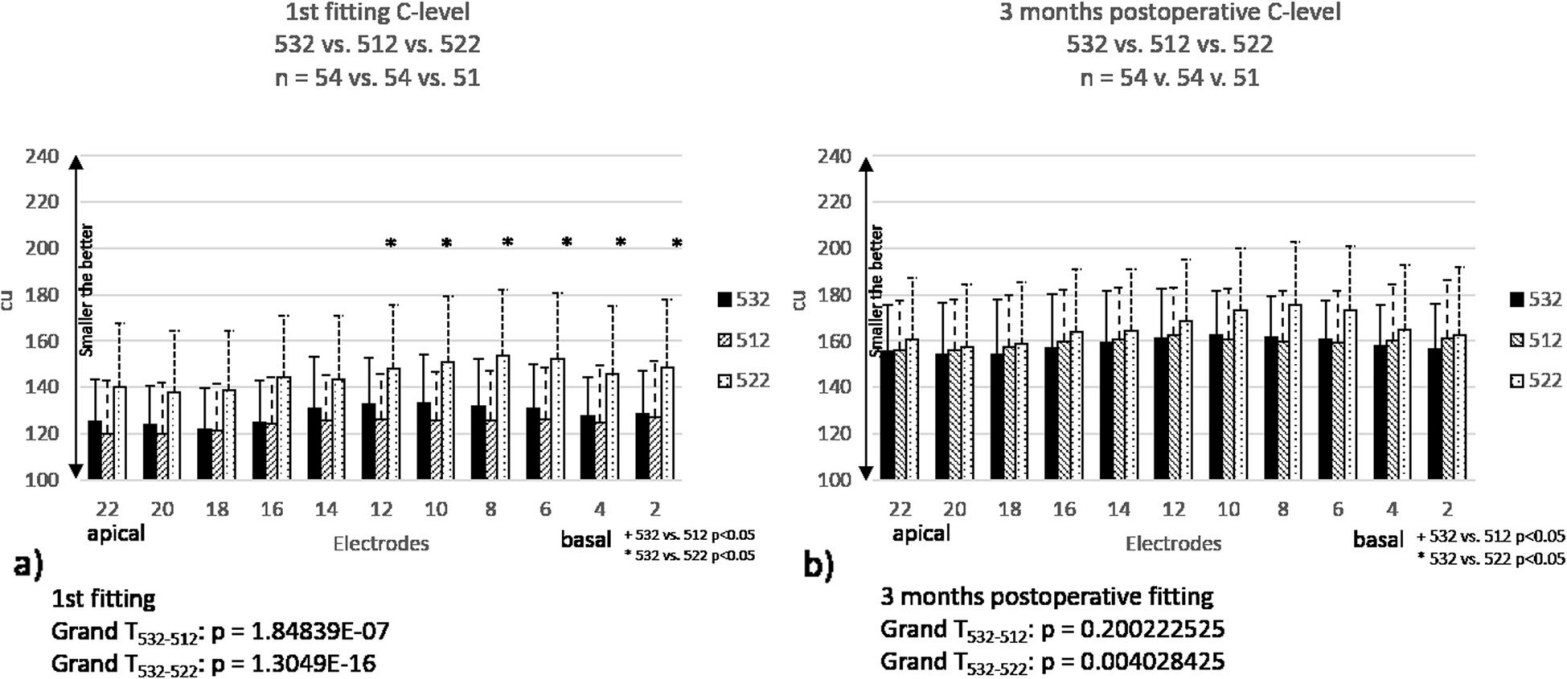
The mean postoperative C-levels in patient groups with different types of CI: Group 532 (*n* = 54, *n* = 54), group 512 (*n* = 54, *n* = 54) and group 522 (*n* = 51, *n* = 51) at first fitting (**a**) and 2-month follow-up fitting (**b**). The “*” stands for a significant difference between groups 532 and 522. Error bars represent the standard deviation (SD). Grand T_532–512_ means statistical comparison between groups 532 and 512. Grand T_532–512_ means statistical comparison between groups 532 and 522

**Fig. 3:**
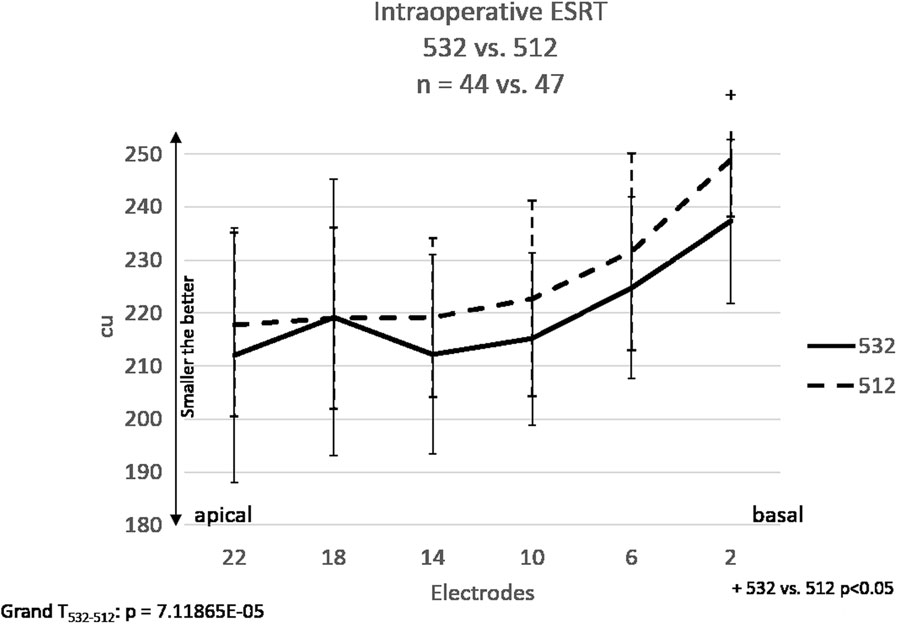
The mean intraoperative ESRT values in patient groups with different types of CIs: group 532 (*n* = 44) and group 512 (*n* = 47). The “+” means a significant difference between groups (**a**) and (**b**). Error bars stand for the standard deviation (SD). „A” stands for Nucleus CI532 and „B″ for Nucleus CI512 implants. Grand T_532–512_ means statistical comparison between groups 532 and 512

Firstly, intraoperative electrical stapedial reflex threshold (ESRT, Fig. 1) and Neural Response Telemetry (T-NRT, Fig. 2), results were compared across implant groups. A stapedial reflex was tested in all subjects in group 532 and 512 and could be elicited in 44 out of 54 cases in group 532 and in 47 out of 54 cases in the control group (group 512). Figure 3 shows that the mean ESRTs were lower in group 532 than in group 512. This difference was significant (t probe: *p* = 0.007) for electrode contact 2. Grand average (all electrodes) statistic calculation (Grand T_532–512_) showed significant differences between groups 532 and 512 (*p* < 0.05).

Intraoperative NRT measurements were performed in all three groups. The neural response threshold was tested in all subjects and could be elicited in 50 out of 54 (group 532), 47 out of 54 (group 512), and 43 out of 51 (group 522) cases. Repeated ANOVA analysis revealed significant difference *p* < 0.05) between the three groups. On examining the significance in pairs, we found that the mean T-NRTs (Fig. 2) proved to be lower in each electrode in group 532 when compared with each control group. The difference was significant in 5 measured electrode contacts when compared with CI522 and 3 measured electrode contacts when compared with CI512 (t-probe: *p* < 0.05). Grand average (all electrodes) statistic calculation (Grand T_532–512_ and Grand T_532–522_) showed significant lower T-NRT values in group 532 compared with the two control groups (*p* < 0.05).

### Postoperative C-levels

**Fig. 4:**
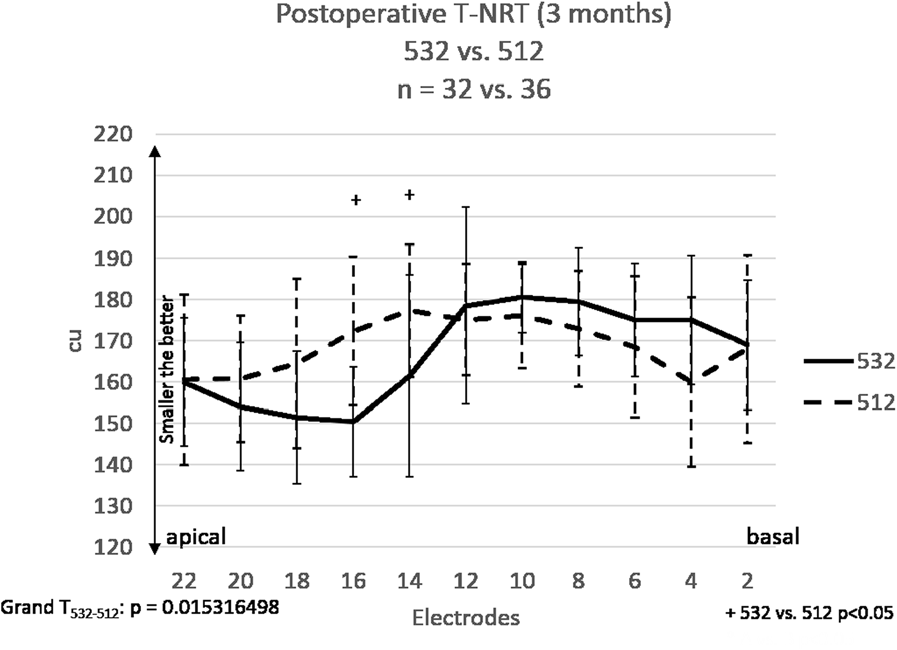
The mean postoperative T-NRT values in subject groups with CI532 (*n* = 32) and CI512 (*n* = 36). The „+” represents a significant difference between groups 532 and 512. Error bars represent the standard deviation (SD). Grand T_532–512_ means statistical comparison between groups 532 and 512

The subjects were scheduled for the first fitting 4 weeks after surgery. C-levels during the first fitting were compared in patient groups with different implants (Fig. 4). No significant difference in mean C-levels was seen on any electrodes between groups 532 and 512, but grand average (all electrodes) statistic calculation (Grand T_532–512_) showed significant differences between the two groups (*p* < 0.05). C-levels were considerably higher on every electrodes in group 522 compared to groups 532 and 512, and the difference was significant for apical electrodes 2 to 12 (*p* < 0.05, Fig. 4)a. Grand average (all electrodes) statistic calculation (Grand T_532–522_) showed significant differences between the groups (*p* < 0.05). However, no significant difference was present on any electrodes in C-levels 2 months after the first fitting, only the grand average statistical analysis (Grand T_532–522_) showed significant differences between groups 532 and 522 (Fig. 4)b.

### Postoperative T-NRT

In group 532 and 512, T-NRT measurements were attempted in all subjects at the two-month follow up fitting and the measurements were successfully carried out in 32 subjects in group 532 and 36 subjects in group 512. The intraoperative electrophysiological measurements could be performed in all subjects under general anesthesia, whereas the postoperative measurements were performed in vigil subjects. In the latter case, some of the subjects complained about unpleasant sound volume before a neural response could have been measured, for this reason the electrophysiological testing cannot be performed.

Figure 4 shows the postoperative mean T-NRT values. The mean T-NRT results in the basal section were lower in group 532 than in group 512. The difference was significant (*p* < 0.05) on two electrodes (No 14 and No 16). Grand average (all electrodes) statistic calculation (Grand T_532–512_) showed significant differences between the groups (*p* < 0.05).

## Discussion

A wide range of cochlear implants with different electrodes are available for rehabilitation of hearing impaired patients with severe to profound sensorineural hearing loss. Hearing rehabilitation outcomes may be influenced by optimizing device and electrode choice for the individual. Several comparative studies have been conducted including electrophysiological (ESRT, NRT) test methods to evaluate the influence of straight and perimodiolar electrode designs and their in-situ characteristics on clinical outcomes [1–3, 25–27]. Our study is unique in that it measured the influence of various electrode designs combined with a common receiver-stimulator upon electrophysiological assessments for a relatively large routinely treated multicenter study cohort. As such, it is the first study to report on the influence of electrode design while using consistent implant receiver-stimulator electronics. The cooperation of the two clinics was established in 2017 with the aim to compare the perimodiolar and the straight electrode arrays. The study clinics followed a standard protocol enabled by the manufacturer’s software, thus a conclusion from their individual results can be made. The results of Hey et al. from their multicenter study on CI532 are in good correlation with our results which proves that our methodology and results are reliable [20].

The Contour Electrode was the first perimodiolar electrode from Cochlear. As reported by researchers, some intracochlear trauma has been associated with its insertion, with a more reliable and less traumatic insertion achieved when deployed using the recommended advance off-Stylet technique [3]. This is largely due to an inherent reduction in intracochlear outer wall force generation when using this technique for this electrode [3].

The Slim Modiolar Electrode is designed for insertion with minimal cochlear trauma. It has the advantage of taking 60% less volume in the scala tympani compared to the Contour Advance Electrode and is therefore placed in a position close to the modiolus. Perimodiolar proximity is an important clinical consideration as Holden et al. [15] concluded, observing that total insertion depth was not associated with better speech discrimination outcomes, however, the distance from the electrodes to the modiolus did indicate a significant influence. The Slim Modiolar electrode array takes a closer position to the modiolus than the Contour Advance electrode array as confirmed by a comparative radiological evaluation [21].

In this retrospective study the data from recipients with the three main types of electrode arrays used in each of the two author implant centers were included. Although the electrode of CI522 was known to take the lateral wall position within the cochlea, the authors’ decided to enroll those subjects who were implanted with CI522 to gain a more detailed overview. Although results of two different implant centers were combined for evaluation, upon review, the authors considered the routine clinical practices employed and device parameters used at each site as sufficiently comparable.

Results from the objective intraoperative measurements indicated that the electrode contacts of the CI532 array were located closer to the modiolus than those of CI512. A previous study found that withdrawal of the stylet in the Contour Advance Electrode resulted in better NRT and ESRT responses, than with the stylet in place. They concluded that this is most probably due to a more favorable position of the electrode array towards the modiolus within the scala tympani once the stylet is removed [26]. In our study, although the mean ESRT was only slightly lower with CI532, the difference was statistically significant at the basal most electrodes tested. However, the mean T-NRT for CI532 was significantly lower than for CI512, especially in the apical-middle section, which is considered to be indicative of closer positioning towards the modiolus. An expected rate of scalar dislocations could be 26% with precurved electrode (i.e. CI512) and 3% with straight electrode (i.e. CI522) with round window insertion technique [28] and this dislocation should have a significant impact on the NRT threshold in the apical part of the electrode. In order to minimize scalar dislocation, the extended round window insertion technique was used. Although the institutional protocols did not include postoperative computed tomography, the results from T-NRT and ESRT, both being constantly higher for CI512 when compared with CI532 and T-NRT being constantly lower for CI512 when compared with CI522 are not indicative of significant dislocations between scalae tympani and vestibuli. The sizeable reduction in both T-NRT and ESRT observed in our study are considered sufficiently large to potentially influence differences in clinical outcomes as observed for subjective comfort level [26, 29].

The surface area of an electrode is inversely proportional with the resistance, thus current is proportional with the surface area. If the electrode with a smaller surface is capable of eliciting the same response it means that it is closer to the stimulated structure. The lower objective electrophysiological thresholds of CI532 suggest that the electrodes are capable of eliciting reflex responses with lower stimulation intensity, resulting from closer proximity to the modiolus.

## Conclusion

Although the Slim Modiolar electrode is significantly thinner than the Contour Advance and similar sized as the Slim Straight electrode array, the Slim Modiolar electrode provides similar or better stimulation productivity compared to Contour Advance and Slim Straight electrodes. The manufacturer’s thinnest electrode array, the Slim Modiolar Electrode takes the position that is closer to the modiolus compared to the Contour Advance Electrode and the Slim Straight Electrode. Our intraoperative and postoperative measurements confirmed this showing that more effective stimulation can be achieved, through the use of the Slim Modiolar Electrode.

## Abbreviations

ANOVA: Analysis of variance
C-level: Comfort threshold
ESRT: Electrically evokedstapedial reflex threshold
NRT: Neural response telemetry
T-level: Electric threshold
T-NRT: Neural response telemetry threshold
